# Effect of Printing Parameters on Tensile, Dynamic Mechanical, and Thermoelectric Properties of FDM 3D Printed CABS/ZnO Composites

**DOI:** 10.3390/ma11040466

**Published:** 2018-03-22

**Authors:** Yah Yun Aw, Cheow Keat Yeoh, Muhammad Asri Idris, Pei Leng Teh, Khairul Amali Hamzah, Shulizawati Aqzna Sazali

**Affiliations:** School of Materials Engineering, Universiti Malaysia Perlis (Unimap), Jejawi 02600, Perlis, Malaysia; ckyeoh@unimap.edu.my (C.K.Y.); asri@unimap.edu.my (M.A.I.); plteh@unimap.edu.my (P.L.T.); amalyhamzah@gmail.com (K.A.H.); shuliz.aqzna@gmail.com (S.A.S.)

**Keywords:** fused deposition modeling, tensile, dynamic mechanical, thermoelectric, infill density, printing pattern

## Abstract

Fused deposition modelling (FDM) has been widely used in medical appliances, automobile, aircraft and aerospace, household appliances, toys, and many other fields. The ease of processing, low cost and high flexibility of FDM technique are strong advantages compared to other techniques for thermoelectric polymer composite fabrication. This research work focuses on the effect of two crucial printing parameters (infill density and printing pattern) on the tensile, dynamic mechanical, and thermoelectric properties of conductive acrylonitrile butadiene styrene/zinc oxide (CABS/ZnO composites fabricated by FDM technique. Results revealed significant improvement in tensile strength and Young’s modulus, with a decrease in elongation at break with infill density. Improvement in dynamic storage modulus was observed when infill density changed from 50% to 100%. However, the loss modulus and damping factor reduced gradually. The increase of thermal conductivity was relatively smaller compared to the improvement of electrical conductivity and Seebeck coefficient, therefore, the calculated figure of merit (ZT) value increased with infill density. Line pattern performed better than rectilinear, especially in tensile properties and electrical conductivity. From the results obtained, FDM-fabricated CABS/ZnO showed much potential as a promising candidate for thermoelectric application**.**

## 1. Introduction

Man-made disruptive actions and limitation of energy sources has driven researchers to actively explore other sources of renewable energy. One of the simplest ways is to recover heat waste is by turning it into electrical energy from temperature gradients through thermoelectric (TE) materials. TE devices possess many unique benefits over other new energy sources, such as no sound pollution, no moving parts, and long operating period, and thus has become one of the best renewable energy technologies to replace traditional energy materials [1].

Conductors and semiconductors are efficient TE materials, but they are associated with issues like high cost of production, scarcity of materials, and toxicity [2]. Therefore, alternative materials, such as polymer composites, are inexpensive and easy to process, and are being studied for TE applications [3,4]. There are several types of reinforcement particles to be incorporated with polymer matrices to obtain better mechanical and TE properties, for example, metal particles, carbon/graphene fibers, metal oxides, silica, and so on [5,6]. Among them, metal oxides have been reported to have great physical and mechanical properties, are low cost and easy to process [5,7], and have therefore drawn much attention. One of the most widely used metal oxide reinforcements is zinc oxide (ZnO), due to its high thermal stability [5,8]. 

The efficiency of a TE material is determined by a dimensionless quantity, called the figure of merit (ZT). ZT = (S^2^σ)T/κ, where S represents the Seebeck coefficient, σ represents the electrical conductivity, T represents the absolute temperature, and κ represents the thermal conductivity. A desirable TE material requires low thermal conductivity, high electrical conductivity and a high Seebeck coefficient [9]. CABS is a mixture of acrylonitrile butadiene styrene (ABS) and carbon black residue. It is electrically conductive, inexpensive, and easy to process. Besides its high thermal stability, ZnO has a higher Seebeck coefficient of −85 μV/K, in comparison to ABS and CABS [10,11]. Furthermore, ZnO particles are relatively easy to disperse as compared to other inorganic particles, such as aluminum oxide (Al_2_O_3_) or titanium dioxide (TiO_2_) [12]. Considering the above-mentioned reasons, CABS/ZnO composite has huge potential as an effective TE material candidate. 

Hot pressing, spark plasma sintering, and melt spinning are among the typical methods to fabricate TE materials. However, these methods are costly, have low flexibility, involve higher temperature, and are more time consuming [13]. Therefore, fused deposition modelling (FDM), which recently has been widely used in various fields, has attracted the attention of many researchers. Several researchers have reported their work in fabricating composite materials via FDM 3D printing method. Dawoud et al. [13] incorporated graphite flakes into ABS filament and printed the composite samples via FDM technique to study the wear and frictional behavior of the composites. He et al. [12] have successfully fabricated TE materials; Bi0.5Sb1.5Te3 samples using 3D printing technique with the samples showed ultralow thermal conductivity of 0.2 W·m^−1^·K^−1^, which is favorable for TE applications. In this work, CABS/ZnO composites were fabricated using a RepRap Mendelmax 1.5 FDM 3D printer. 

For TE generators, the individual TE elements are subjected to significant mechanical stresses under in-service conditions. Therefore, a promising TE material needs to be good in TE properties, as well as in its mechanical properties. But, the parts made through FDM are usually said to be inferior to those that are made from conventional processes, in terms of both strength and accuracy [14]. In another study by Dawoud et al. [15], FDM, in contrast to injection molding, was studied to investigate the effect of processing techniques on the mechanical behavior of pure ABS. ABS samples prepared by injection molding generally performed better in the conducted tests if compared to those of 3D printing. However, results showed that an adequate selection of FDM parameters was able to reach mechanical properties comparable to those of injection molded parts in both static and dynamic loading modes. This has proven that the properties of the fabricated part could be enhanced by altering and optimizing the FDM printing parameters. Therefore, one of the main focuses of this study is to explore the interrelationship between FDM process parameters and their impact on the material properties.

Among various parameters, infill density and printing pattern were found to greatly influence the material properties of the printed samples [16,17]. In this paper, the effect of these two parameters on tensile properties were studied. Very limited literature has been reported on 3D printed polymer composite focusing on the effect of printing parameters on both the dynamic mechanical and TE behavior of the material. Therefore, this research aims to explore the TE behavior of manufactured composites, so as to provide better understanding of them. 

## 2. Materials and Methods 

### 2.1. Raw Materials

The raw materials used were ABS filament (MakerBot Industries, New York, NY, USA), CABS filament (YOYI, Guangdong, China), and ZnO powder with ≥99% purity (HmbG Chemicals, Johor, Malaysia). Both ABS and CABS filaments with the diameter of 1.75 mm were employed as the feedstock for the FDM process. The purchased CABS filament consists of a mixture of ABS and blends of carbon black with the electrical resistivity of 128 ohm/cm. Based on the previous investigations, the amount of the carbon black residue determined from thermogravimetric analysis (TGA) was 28 wt %. Filler precoating was conducted before 3D printing process to help improve the interfacial bonding between ABS matrix and ZnO fillers. The precoating process involved mixing 20 g of ABS with 250 mL acetone, followed by magnetically stirring the solution until ABS was fully dissolved. ZnO powder was then added into the ABS/acetone solution with the ratio of 1:1. Mechanical grinding was done using mortar and pestle for 30 min to deagglomerate wetted powder. The powder was then left dried, at room conditions, for 2 h. 

### 2.2. Sample Fabrication via FDM 3D Printing

**Figure 1:**
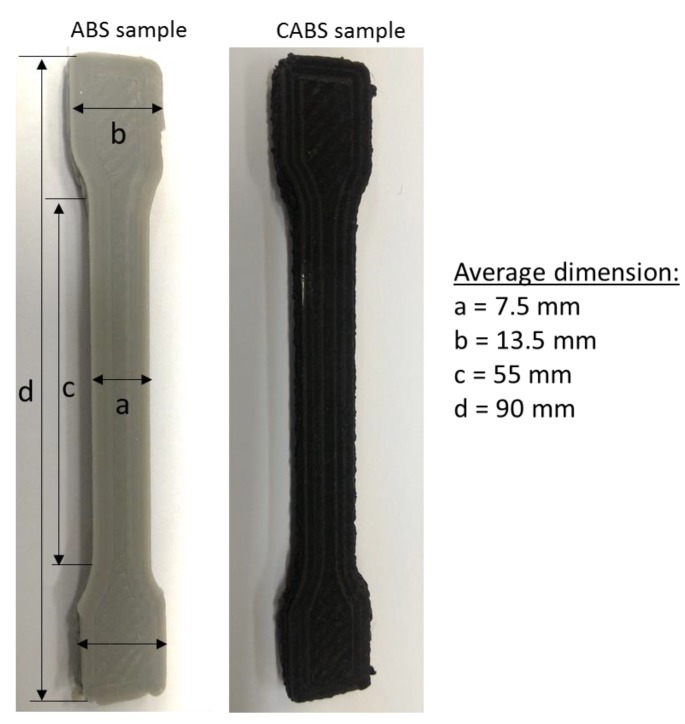
ABS and CABS samples.

**Figure 2:**
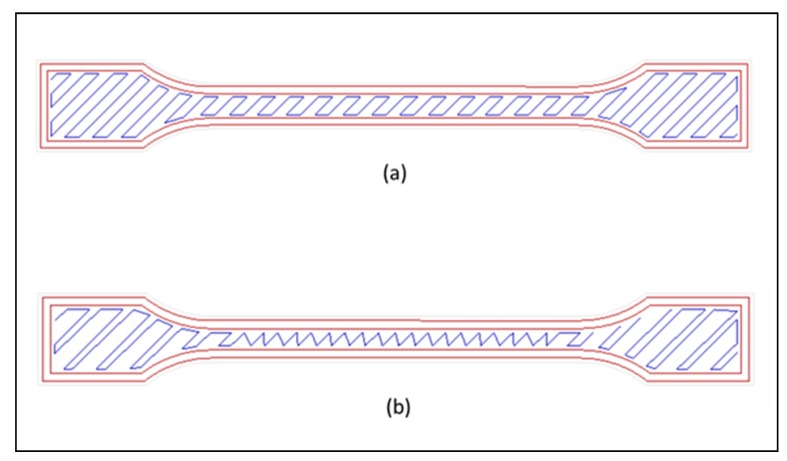
Different printing patterns (Image from Slicer software, [19]). (**a**) Rectilinear and (**b**) line.

Samples were fabricated using RepRap Mendelmax 1.5 FDM desktop 3D printer [18] (Maker’s Tools Works, Oklahoma City, OK, USA). During the printing process, filaments with diameter of 1.75 mm were heated, and 14 wt % ZnO powder was dispensed from the modified dispenser into the polymer matrix. ZnO content of 14 wt % was selected, since parts fabricated by such a content have led to overall outstanding mechanical properties, as shown in previous study. The samples were fabricated in line and rectilinear pattern at 45° raster angle by varying the infill density (50%, 75%, and 100%). Sample was printed in dumbbell shapes, as shown in Figure 1, for tensile testing purposes. Rectilinear pattern creates a rectangular network with 45° congruent to each other, while line pattern forms infill patterns with linear connections between the walls, as shown in Figure 2. The temperature of extruder and bed was fixed at 230 °C and 90 °C, respectively. 

### 2.3. Characterization Methods and Measurements

**Figure 3:**
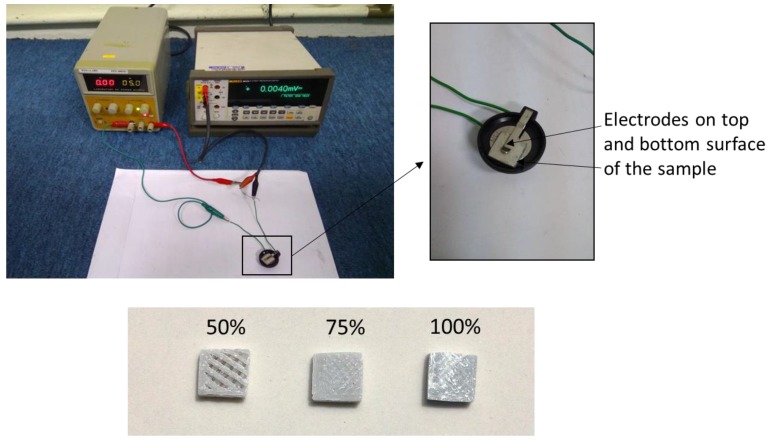
Electrical conductivity measurement setup.

Tensile testing was carried out on the printed samples according to ASTM D638 using Universal Instron machine (Instron, Norwood, MA, USA), which was set at a crosshead speed of 5 mm/min [20]. The average result was taken of five samples for each formulation. Dynamic Mechanical Analysis (DMA) measurements were performed in Seiko DMS 210 (Seiko Instruments Inc., Chiba, Japan) in tension mode at 1 Hz. The testing was carried out from 30 to 150 °C at a rate of 5 °C/min under constant nitrogen flow. Dynamic storage modulus, loss modulus, and damping factor were obtained from the results. Electrical conductivity measurement was carried out using a two-probe method with a Keithley’s source measure unit (Model SMU 236) (Tektronix, Beaverton, OR, USA) at room temperature, as shown in Figure 3. Samples were cut into dimensions of 1 cm (W) × 1 cm (L) × 0.4 cm (H), and polished on both sides into a thickness/height of 2 mm. 

Prior to measurement, a small amount of silver paste applied in a thin layer covered the top and bottom surface of the sample to prevent contact resistance between the samples and electrodes. The electrodes were then placed on top and bottom surface of the samples. The electrical conductivity was determined by Ohm’s law, as illustrated in Equation (1).

(1) $$\sigma =\;\frac{I}{\mathrm{RA}}$$

where σ is electrical conductivity (S/cm), R is resistance (Ω), I is thickness of material (cm), and A is cross-sectional area (cm^2^). Experimental thermal conductivity was determined using temperature differential method in the temperature range of 25–95 °C according to ASTM C177-13 [21]. The thermal conductivity was calculated based on Fourier’s law of heat conduction, as shown in Equation (2).

(2) $$\kappa =\;\frac{Q\;x}{A\;\Delta T}$$

where κ is thermal conductivity (W/mK), Q is heat flow (W), x is thickness of material (m), A is cross-sectional area (m^2^), and ∆T is temperature difference (K). The Seebeck coefficient, also called the thermopower, measures the voltage generated between two points in the material per unit temperature difference between these points. Its value is expressed in the unit of μV/K. Seebeck coefficient was calculated using Equation (3), where ∆V was the voltage produced across the sample due to temperature difference ∆T.

(3) $$S=\frac{\Delta V}{\Delta T}$$

where S is Seebeck coefficient (V/K), ∆V is voltage produced (V) and ∆T is temperature difference (K). The efficiency of a thermoelectric material (ZT) is determined by a dimensionless quantity called the figure of merit. The ZT value was calculated based on Equation (4).

(4) $$\mathrm{ZT}=\frac{{\sigma S}^{2}T}{\kappa }$$

where Z is figure of merit, σ is electrical conductivity (S/cm), S is Seeebeck coefficient (V/K), T is absolute temperature, and κ is thermal conductivity (W/mK).

## 3. Results and Discussion

### 3.1. Effect of Printing Parameters on Tensile Properties

**Figure 4:**
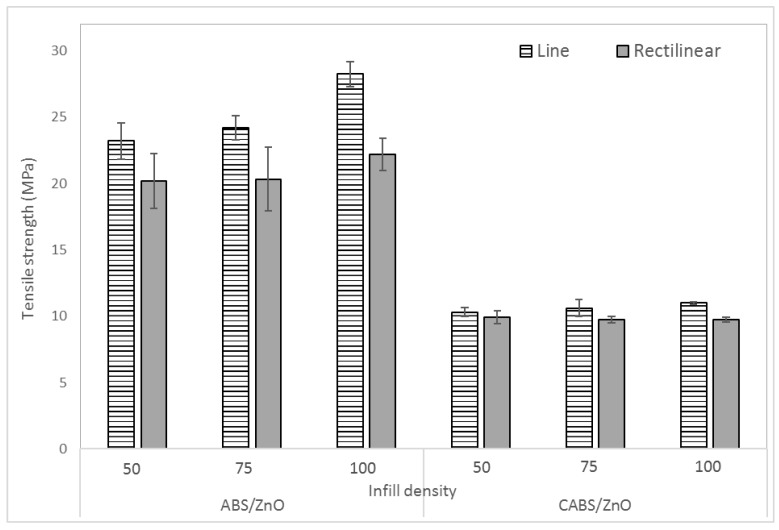
Effect of infill density and printing pattern on tensile strength of ABS/ZnO and CABS/ZnO samples.

Figure 4 shows the effect of infill density and printing pattern on the tensile strength of the composites. For both line and rectilinear samples, the tensile strength increased gradually as the infill density increased. The tensile strength for ABS/ZnO line samples were 23.3, 24.19, and 28.24 MPa for the infill density of 50%, 75%, and 100%, respectively. For CABS/ZnO line samples, the tensile strength improved 6.3% to 10.31 MPa when infill density changed from 50% to 100%. The tensile strength for ABS/ZnO rectilinear samples were 20.21, 20.32, and 22.19 MPa for the infill density of 50%, 75%, and 100%. For CABS/ZnO rectilinear samples, only minor improvement was observed (~2%) when infill density changed from 50% to 100%. Maximum tensile strength of 28.24 MPa was observed for ABS/ZnO sample printed with the combination of line pattern and 100% infill density. At 100% infill density, there was no gap between printed lines, and each layer started to form, bonding with the next layer. The ability of printed layers to deform and absorb the stress before a break in the bonds between different printed layers increased, and thus, the tensile strength of the sample increased [17,18]. When the infill density decreased, the gap between printed strands became larger, and the bonding between layers became weaker, therefore tensile strength decreased [16]. The tensile strength of the ABS parts was found in the range of 20–28 MPa. Based on the study of Dawoud et al., the tensile strength of 3D printed ABS samples was in the range of 65–72% of injection molded parts. They reported the injection molded part with tensile strength of 33–38 MPa. This result was in good agreement with the findings reported by Dawoud et al. [15]. However, the tensile strength of CABS composites was much lower than that of ABS composites and revealed the poor adhesion between carbon black, ABS, and ZnO.

As shown in Figure 4, the tensile strength of rectilinear samples was always lower compared to line samples, probably due to the randomness in printing orientation. Line pattern formed infill pattern with linear connections between the walls, while rectilinear pattern created a rectangular network with 45° congruent to each other. Rather than making multiple 90° turns to create a cross-hatched pattern for rectilinear samples, the printer made straight lines across the entire length of the print for line samples, and therefore, more consistent extruder motion was seen while printing line samples [22,23]. Hernandez et al. studied infill density as one of the factors to cause 3D printing extrusion problems. They reported that by increasing the amount of material the printer must deposit, which means increasing the infill density, can help to alleviate extrusion problems. Besides that, printing patterns which exhibited more consistent extruder motion allowed for more consistent adhesion between layers [23]. This is in good agreement with the finding where line samples have higher tensile strength compared to rectilinear samples, which induced less consistent extruder motion during printing process.

**Figure 5:**
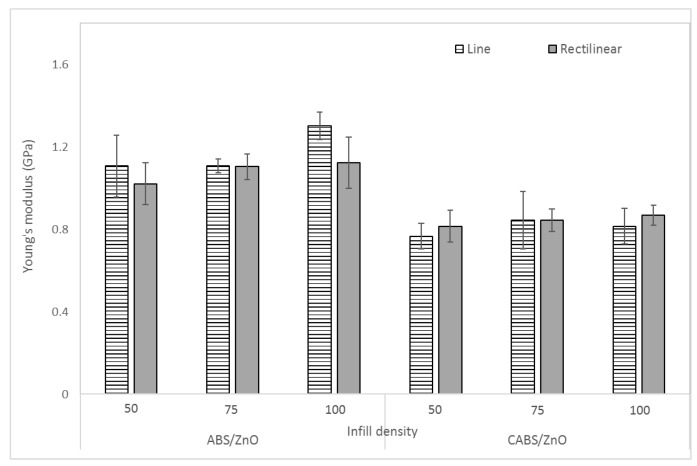
Effect of infill density and printing pattern on Young’s modulus of ABS/ZnO and CABS/ZnO samples.

Figure 5 shows the effect of infill density and printing pattern on Young’s modulus of the composites. The combination of line pattern with 100% infill density showed the highest Young’s modulus, with the value of 1.3 GPa. For ABS/ZnO, the modulus value increased 15% from 1.11 to 1.3 GPa when infill density changed from 50% to 100%. Whereas for CABS/ZnO, the modulus increased 7.4% from 0.81 to 0.87 GPa when infill density increased from 50% to 100%. Stiffness of the samples increased with the infill density, so as the Young’s modulus [24]. This was due to the ability of filament between different layers to resist deformation having improved, which resulted in greater stiffness when the infill density increased. Several papers in public literature have reported similar findings [17,19,22]. Among them, Fernandez et al. [16] investigated the tensile behavior of ABS samples printed in three different printing patterns, which were line, rectilinear, and honeycomb. The infill density of 20%, 50%, and 100% were evaluated. The tensile modulus of all patterns increased when infill density changed from 20% to 50%. The increase was even more significant between 50% and 100%, which was due to the improvement of the capability to deform and absorb the stress before a break by creating bonds between different layers of infill fibers. 

Between the two different printing patterns, line samples were reported with higher modulus value than rectilinear samples. Rectilinear samples with less consistent adhesion layers were less stiff, and therefore obtained slightly lower Young’s modulus value compared to line samples. Besides that, the raster angle of rectilinear pattern [−45°, 45°] is smaller compared to line pattern. In a work conducted by Kulkarni et al. [25], the stiffness of samples with five different combinations of raster angle, [0°, 90°], [15°, −75°], [30°, −60°], [45°, −45°], and [60°, −30°], were determined. Among all the different raster angles used, the [45°, −45°] had the lowest stiffness value in the testing direction. Optimum stiffness means the highest tensile modulus were observed at [0°, 90°] raster angle. This indicated that if a greater stiffness is required, then the part should be oriented so that the material axis coincides with that direction. [25]. The finding was similar to the results in this study where rectilinear pattern [−45°, 45°] reported lower tensile modulus value compared to line pattern.

**Figure 6:**
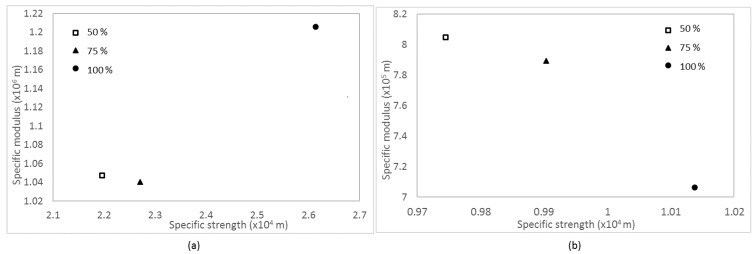
Effect of infill density on specific strength and modulus of (**a**) ABS/ZnO and (**b**) CABS/ZnO samples.

Figure 6 shows the effect of infill density on specific strength and modulus of the samples. For ABS samples, samples with 100% infill density appeared to have higher specific modulus and strength values than those with 50 and 75% infill density. However, the trend was opposite for CABS samples. Samples printed with 50% infill density were observed to have higher specific modulus and strength values, followed by those printed with 75 and 100% infill density. Comparing between ABS and CABS, the specific strength and modulus value of ABS composite was higher, and indicated that it has light weight with high modulus and strength.

**Figure 7:**
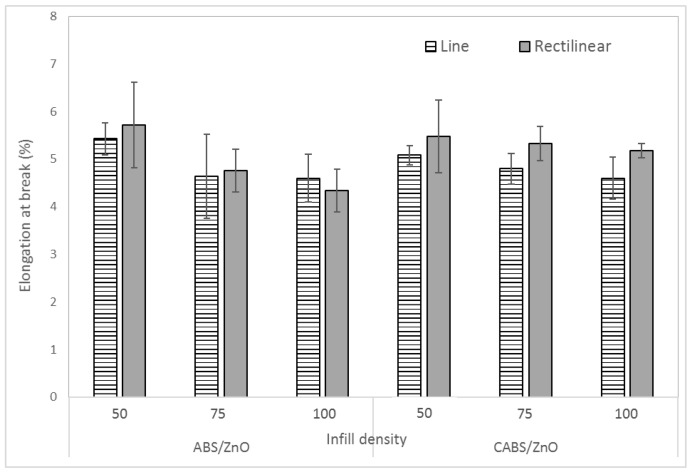
Effect of infill density and printing pattern on elongation at break of ABS/ZnO and CABS/ZnO samples.

Figure 7 shows the effect of infill density and printing pattern on the elongation at break of the composite samples. The elongation at break for ABS/ZnO line samples were 5.43%, 4.64%, and 4.6% for the infill density of 50%, 75%, and 100% respectively. For CABS/ZnO line samples, the elongation at break reduced from 5.08% to 4.6% when infill density increased from 50% to 100%. The elongation at break for ABS/ZnO rectilinear samples were 5.72%, 4.76%, and 4.34% for the infill density of 50%, 75%, and 100% respectively. For CABS/ZnO rectilinear samples, the elongation at break reduced from 5.48 to 5.18 when infill density increased from 50% to 100%. The increase of infill density improved the stiffness of the samples due to better bonding between printed layers, and thus, reduced the elongation at break of the composites [24]. Rectilinear samples which were more flexible elongated more than line samples. 

### 3.2. Effect of Printing Parameters on Dynamic Mechanical Properties

**Figure 8:**
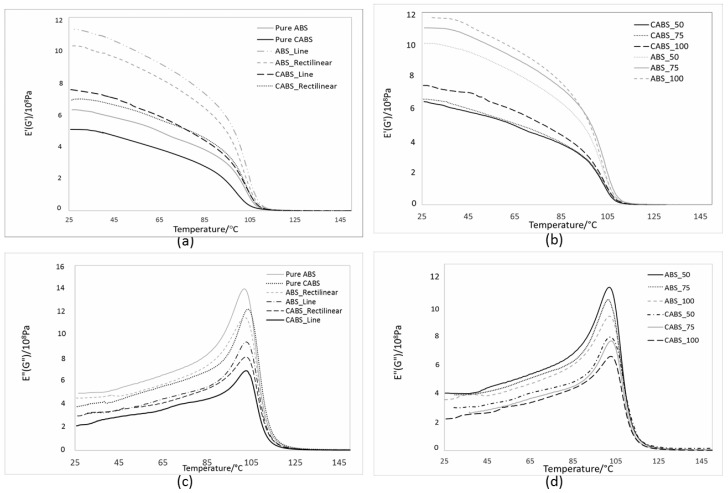

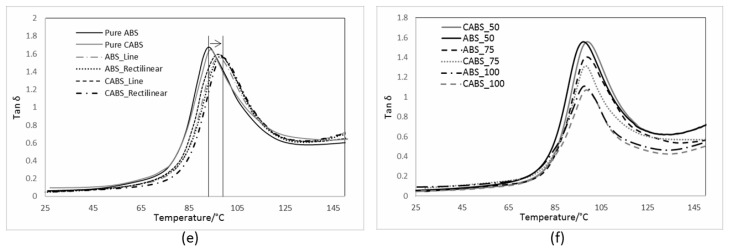
Effect of infill density and printing pattern on dynamic mechanical properties. (**a**,**b**) Storage modulus (E’), (**c**,**d**) loss modulus (E’’), and (**e**,**f**) damping factor.

Figure 8a,b show the dynamic storage modulus, E’, of the composites at different printing pattern and infill density, respectively. E’ measures the stiffness and elasticity of a material [24,25]. The E’ curve underwent the characteristic steep decrease from 30 °C to 100 °C, due to the increase in the molecular mobility of the polymer chains when temperature increased [26,27]. The E’ values of composites are higher than that of pure ABS and CABS at the same temperature, owing to the stiffening effect resulting from ZnO filler addition. By adding in fillers that having good interfacial adhesion with matrix, the storage modulus will be improved [28]. However, ineffective stress transfer between fillers and matrix occurred when the spacing between filament strands was greater. The reduction of the stiffening effect of fillers resulted in lower E’ value. When the infill density increased, a close raster and deposited fibers were generated, which led to a denser structure and improvement in E’ of the printed parts [29]. Rectilinear samples showed a slightly lower storage modulus value at all temperatures below glass transition temperature, Tg, compared to line samples, due to less stiff structure.

Figure 8c,d show the loss modulus, E”, of composites at different printing pattern and infill density, respectively. Loss modulus is the ability of the material to lose energy, which is inversely proportional to storage modulus [27]. More energy is dissipated due to stiffness reduction, and therefore, loss modulus is maximum for samples which are less stiff. Therefore, comparing between line and rectilinear pattern, loss modulus was greater for rectilinear samples which were less stiff. Loss modulus peak, as well as the Tan δ peak, were found to be shifted towards higher temperatures for both ABS and CABS composites as compared to pure ABS and CABS, which indicated higher thermal stability of composites as a result of filler addition. After the maximum peak, loss modulus decreased due to the increase of polymer chain mobility. Both the peak of loss modulus and Tan δ can be used to determine the glass transition (Tg) of the material, and there would be variation on both values. In our study, Tan δ was used to determine the Tg. The Tg of pure ABS and CABS was in the range of 94–95 °C, and increased to 101–103 °C when filler was added in. No significant impact was observed on the Tg for printing pattern and infill density change. Increasing Tan δ means the material has more energy dissipation potential, whereas decreasing Tan δ means when load is applied, the material has more potential to store the load, rather than dissipating it [28,29]. Results in Figure 8e showed Tan δ decreased with infill density. In Figure 8f, rectilinear samples showed comparable damping with line samples.

### 3.3. Effect of Printing Parameters on Thermoelectric Properties

**Figure 9:**
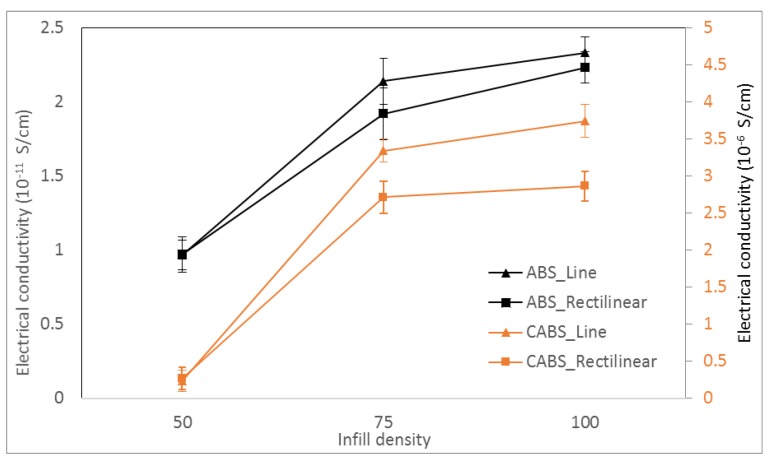
Electrical conductivity of ABS and CABS composites with different infill density and printing pattern.

Figure 9 shows the electrical conductivity of ABS and CABS composites with different infill density and printing pattern, respectively. Pure ABS has the electrical conductivity of 9.16 × 10^−12^ S/cm, whereas pure CABS has higher electrical conductivity, which is 1.312 × 10^−7^ S/cm. This is due to the carbon black content in CABS, which has electrical conductivity as high as 5.58 S/cm. We found that higher infill density led to higher electrical conductivity. The conductivity of ABS line sample was 9.64 × 10^−12^ S/cm, and increased to 2.14 × 10^−11^ S/cm and 2.33 × 10^−11^ S/cm, with increasing infill density from 50% to 100%. The conductivity of ABS rectilinear sample was 9.71 × 10^−12^ S/cm at 50% infill density and improved to 2.23 × 10^−11^ S/cm at 100% infill density.

**Figure 10:**
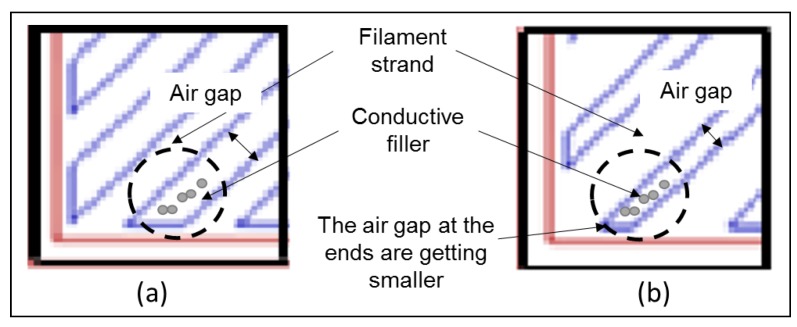
Air gap between filament strands. (**a**) Rectilinear; (**b**) Line (Illustration of toolpath in square sample).

For CABS, the conductivity of line sample was 2.32 × 10^−7^ S/cm, and increased to 3.74 × 10^−6^ S/cm with the increase of infill density from 50% to 100%. For rectilinear sample, conductivity was 2.64 × 10^−7^ S/cm at 50% infill density, and increased to 2.86 × 10^−6^ S/cm at 100% infill density. In general, electrical conductivity increased with infill density due to the formation of more conductive pathways between ZnO fillers and carbon black within the filament strands when the filaments were deposited closer together. Line samples were reported with higher electrical conductivity compared to rectilinear samples. At 50% infill density, the conductivity difference between rectilinear and line pattern was insignificant. However, there was noticeable difference at 75% infill density, possibly due to the air gap difference between two different patterns. Figure 10 shows the air gap between rectilinear and line pattern. The air gap of line pattern at both connecting ends are getting smaller when it approached the wall, and increased the possibility of the conductive fillers within the air gap to form conductive path across the materials. Therefore, a huge step-up was observed when density changed from 50% to 75%. When density changed to 100%, only a minor increase in conductivity was observed, which indicated optimum conductivity has reached at 75% infill density. For CABS, the difference of conductivity at 100% infill density between line and rectilinear was more significant. That could be due to higher porosity within rectilinear CABS samples. Printing pattern which exhibited less consistent extruder motion, which was rectilinear pattern, led to less consistent adhesion between layers; therefore, more voids/pores formed within rectilinear samples. Besides that, the formation of voids/pores in CABS samples was also contributed to by the formation of agglomerations, due to poor adhesion between fillers and matrix. 

**Figure 11:**
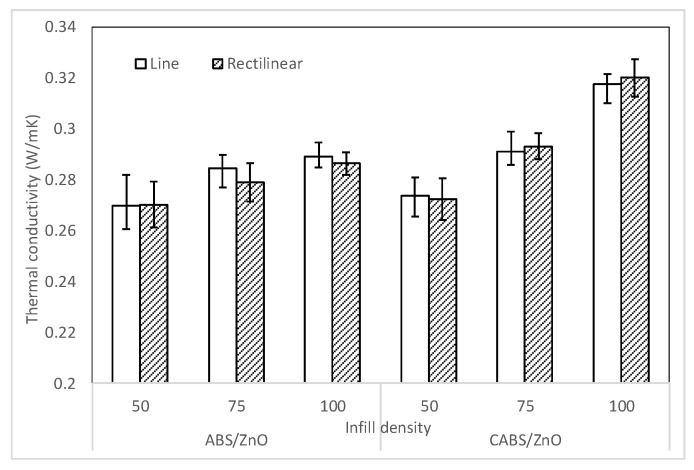
Thermal conductivity of ABS and CABS composites with different infill density and printing pattern.

Figure 11 shows the thermal conductivity of ABS and CABS composites with different infill density and printing pattern. Thermal conductivity of CABS/ZnO was found higher than that of ABS/ZnO, due to the presence of intrinsically high thermal conductivity carbon black [30]. The thermal conductivity of ABS line and rectilinear samples were 0.2697 and 0.2701 W·m^−1^·K^−1^. The conductivity values increased 7% and 5.6% to 0.2892 and 0.2863 W·m^−1^·K^−1^, respectively with increasing infill density from 50% to 100%. Similar trend was observed for CABS composites. The conductivity of line and rectilinear sample increased 13.8% and 15% to 0.3174 and 0.32 W·m^−1^·K^−1^ at 100% infill density. The thermal conductivity was observed to be improved with infill density, due to better heat transfer when the air gap between filament strands was getting smaller. The thermal conductivity values for both line and rectilinear samples were comparable, indicated the printing pattern has no impact on the thermal conductivity of the parts.

**Figure 12:**
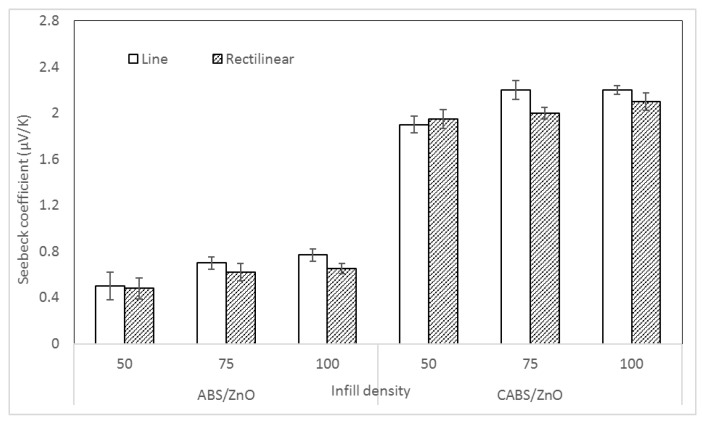
Seebeck coefficient of ABS composites with different infill density and printing pattern.

Seebeck coefficient of ABS and CABS composites with different infill density and printing pattern is illustrated in Figure 12. There was 35% and 26% of improvement when infill density increased from 50% to 100% for ABS line and rectilinear samples, respectively. For CABS line and rectilinear sample, the Seebeck coefficient increased 43% and 24% to 2.2 and 2.1 μV/K. The increase of Seebeck coefficient may be attributed to the larger number of layer interfaces between polymer matrix and fillers, which increased the carrier mobility, and thus, improved the Seebeck coefficient [31,32]. Similar to the electrical conductivity, line samples were reported with higher Seebeck coefficient than the rectilinear samples.

**Figure 13:**
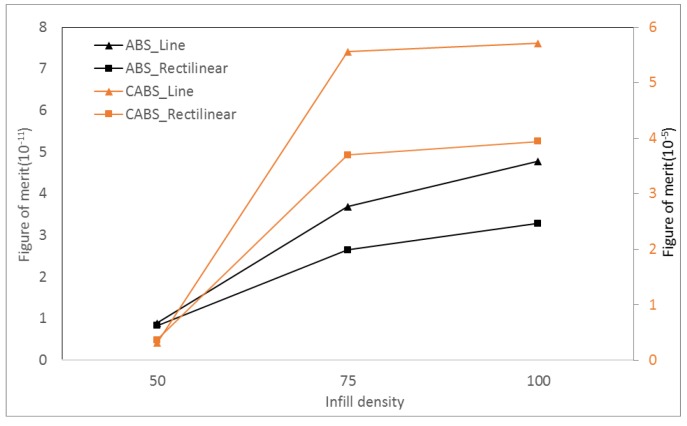
Figure of merit of ABS and CABS composites with different infill density and printing pattern.

Figure 13 shows the figure of merit of ABS and CABS composites with different infill density and printing pattern. The increase of thermal conductivity was relatively smaller compared to the improvement of electrical conductivity and Seebeck coefficient, therefore, the overall ZT value increased with infill density. ZT value of ABS line sample printed in 50% infill density was 3.7 × 10^−11^. By increasing the infill density to 100%, the ZT value increased to 4.8 × 10^−11^. For rectilinear sample, the ZT values were 8.3 × 10^−12^, 2.7 × 10^−11^, and 3.3 × 10^−11^ at infill density of 50%, 75%, and 100%, respectively. On the other hand, the ZT value of CABS line samples were improved from 3.1 × 10^−6^ to 5.7 × 10^−5^ with increasing infill density from 50% to 100%. A similar trend was seen on CABS rectilinear sample, where there was 90% of ZT value improvement when infill density increased from 50% to 100%. The calculated ZT results followed the improvement trend of electrical conductivity, in which line samples performed better than rectilinear samples. 

## 4. Conclusions

In this study, the effects of printing parameters, including infill density and printing pattern, on the tensile, dynamic mechanical, and thermoelectric properties of FDM-fabricated CABS/ZnO composites were investigated. The tensile strength of CABS composites was lower compared to ABS composites, and insensitive to the change of infill density. Young’s modulus increased when infill density increased, due to formation of bonding between printed layers resulting in higher stiffness. However, the stiffness improvement reduced the elongation at the break of the composites. ABS/ZnO printed with 100% infill density exhibited the highest specific strength, and modulus value indicated that it has light weight, with high modulus and strength. Dynamic storage modulus improved, while the loss modulus and damping factor reduced gradually with infill density. Effective stress transfer between fillers and matrix resulted in higher storage modulus value, but lower loss modulus, as less energy was dissipated due to stiffness improvement. Infill density brought positive impact on electrical conductivity, Seebeck coefficient, and thermal conductivity. However, a relatively small change in thermal conductivity when compared with their electrical conductivity and Seebeck coefficient resulted in high ZT. Line pattern, which exhibited more consistent extruder motion during printing process, possesses better tensile properties. Samples printed with line pattern were stiffer, and therefore have higher storage modulus, but lower loss modulus and damping factor. For thermoelectric properties, the calculated ZT results followed the improvement trend of electrical conductivity, in which line samples performed better than rectilinear samples.
